# Prognostic value of the number of biopsied sentinel lymph nodes for Chinese patients with melanoma: A single‐center retrospective study

**DOI:** 10.1002/cnr2.1958

**Published:** 2023-12-26

**Authors:** Tu Hu, Yu Xu, Wangjun Yan, Chunmeng Wang, Wei Sun, Yunyi Kong, Yong Chen

**Affiliations:** ^1^ Department of Musculoskeletal Surgery Fudan University Shanghai Cancer Center Shanghai China; ^2^ Department of Oncology, Shanghai Medical College Fudan University Shanghai China; ^3^ Department of Pathology Fudan University Shanghai Cancer Center, Fudan University Shanghai China

**Keywords:** lymph node metastasis, melanoma, prognosis, sentinel lymph node biopsy

## Abstract

**Background:**

Sentinel lymph node biopsy (SLNB) helps to determine accurate pathological stages and facilitates strategies for regional disease control in melanoma. However, whether the number of biopsied sentinel lymph nodes (SLNs) influences the patients' survival is rarely investigated.

**Methods:**

Acral or cutaneous melanoma patients with no history of nodal disease who received SLNB in Fudan University Shanghai Cancer Center (FUSCC) from January 1, 2017, to December 31, 2021 were retrospectively enrolled. Clinicopathological variables including Breslow index, ulceration, number of positive SLNs, SLN/non‐SLN status were analyzed. The pathologic nodal (pN) stage and pathological stage were defined.

**Results:**

A total of 381 eligible patients were enrolled in this study, of whom 132 (34.7%) patients were diagnosed with SLN‐positive. The median number of biopsied SLNs was 2 (range: 1 to 20). Different numbers of biopsied SLNs did not influence the release‐free survival (RFS) of the general patients. However, patients with >2 SLNs had a longer RFS than those with 1–2 SLNs in T4, N1a group and those who rejected complete lymph node dissection (CLND).

**Conclusions:**

In patients with T4 melanomas, N1a melanomas and those that did not undergo a CLND, the prognosis of those with three or more SLNs retrieved seemed to be improved.

## 1 INTRODUCTION

Sentinel lymph node biopsy (SLNB) has become a standard treatment for clinically node‐negative cutaneous and acral melanoma patients. To detect micro‐metastasis in the regional lymph node basin in a timely manner, SLNB helps to identify the pathological stage precisely and thereby facilitates therapeutic strategy decisions and improves regional disease control. It also aids to pick out potential patients who might benefit from postoperative adjuvant therapy and improve the prognosis ultimately.^1^ The metastatic status of sentinel lymph nodes is an independent prognostic factor of melanoma, as well as primary tumor thickness.^2^, ^3^ SLNB is suggested for primary melanoma patients with Breslow thickness more than 0.8 mm, or less than 0.8 mm with risk factors, for instance, regression, ulceration, neurovascular invasion, and young age.^3^ Our previous study also showed that non‐SLN positivity was also negatively correlated with disease‐free survival (DFS) and overall survival (OS) in patients who received completion lymph node dissection (CLND) after identification of one positive SLN.^4^ In addition, numerous studies have implicated that tumor burden in SLN, such as maximum diameter and location of micro‐metastatic lesions, number of metastatic foci, or presence of lymph node extracapsular extension, has significant effects on postoperative outcomes for patients with melanoma.^5^, ^6^, ^7^, ^8^, ^9^ Macro‐metastasis of SLN invasion was associated with higher risk of non‐SLN involvement and always indicated a poor prognosis with respect to micro‐metastases.^10^

However, few studies have concentrated on the prognostic role of other indicators of SLNB. One major concern is the accuracy of SLNB, which depends on the lymphatic mapping and adequacy of nodal biopsy. The number of biopsied SLNs was rarely discussed in previous studies, because SLNB is generally considered as a minimally invasive procedure to prevent limb swelling and numbness. On the other hand, more than one node is detected in clinical practice during SLNB. The biopsy of all identified SLNs might lead to more accurate pathological stagging. According to the stagging criteria, more regional nodes involved with metastatic melanoma is always classified as higher stage and is related to bad prognosis. However, Jakub et al. found if the positive lymph nodes is confined to the SLNs without positive non‐SLNs, the number of regional lymph nodes involved with metastatic disease does not affect DFS and OS.^11^ In addition, inadequate number of SLNs was found to increase the positivity rate in non‐SLNs.^12^ Thus, except for a minimal requirement on the number of lymph nodes evaluated after CLND,^13^ it is still essential to indicate minimal SLNs that should be biopsied during SLNB. Therefore, the present single‐center retrospective study aimed to further investigate the prognostic value of the number of biopsied SLNs for Chinese patients with melanoma.

## 2 PATIENTS AND METHODS

Primary cutaneous or acral melanoma patients with no clinical nodal disease, who underwent SLNB at Fudan University Shanghai Cancer Center (FUSCC; Shanghai, China) from January 1, 2017, to December 31, 2021 were retrospectively enrolled in the study. Patients with incomplete medical data or with a follow‐up of less than 6 months were excluded from the study. Clinicopathological variables, including gender, age, Clark level, Breslow thickness, ulceration, number of positive SLNs, SLN/non‐SLN status, and adjuvant therapy, were recorded. The pathologic nodal (pN) stage and pathological stage of patients were defined based on the 8th edition of the American Joint Committee on Cancer (AJCC) staging manual.^14^

### 2.1 SLNB

SLNB was routinely performed under lymphatic mapping through lymphoscintigraphy by technetium‐99 sulfur colloid, methylene blue dye, or both. The pathological examination of resected SLN was described in our previously published study.^15^ Briefly, each of SLNs was dissected at 3 mm thickness or along the longest axis on the largest surface, fixed by 3.7% neutral formaldehyde, then dehydrated, conventionally embedded into paraffin and finally stained with hematoxylin and eosin (HE). The metastatic status of SLNs was also determined by immunohistochemical staining of HMB45, S‐100, SOX10 and Melan A. The antibodies against S‐100 and Melan A were purchased from Dako Co., Ltd. (Copenhagen, Denmark). The antibody against HMB45 was purchased from MaiXin Biotechnologies Co., Ltd. (Fuzhou, China). The antibody against SOX10 was purchased from Gene Tech Co., Ltd. (Shanghai, China). Each section was observed under a light microscope by two pathologists. The majority of patients with metastatic SLNs received CLND within 1 month.

### 2.2 Follow‐up

Patients were assessed through clinical examinations, such as routine physical checkup, ultrasound, computed tomography and/or magnetic resonance imaging every 3 months for the first 2 years, every 6 months for 3–5 years, and then annually. Patients were followed up through reexamination or telephone follow‐up until death or December 31, 2021. The survival of patients was censored at the date of the last follow‐up (December 31, 2021). Relapse‐free survival (RFS) was defined as the interval between radical surgery and the first recurrence or distant metastasis. Recurrence or metastasis was confirmed by pathology or imaging follow‐up.

### 2.3 Statistical analyses

Categorical variables were described by the frequency and component ratio. The Pearson's chi‐squared test or the Fisher's exact test was used for univariable analysis between patients with 1 to 2 biopsied SLNs and those with more than 2 biopsied SLNs. The Kaplan–Meier method and the log‐rank test were adopted to describe and compare the impacts of some prognostic factors (SLN‐positivity, acral subtype, N1a stage, T4 stage and SLN‐positivity without CLND) on RFS between these two groups. *p*‐Values less than .05 were considered statistically significant. The statistical analysis was performed using SPSS 22.0 software (IBM, Armonk, NY, USA). This study was approved by the Ethics Committee of FUSCC, and every participant signed an informed consent form before enrollment.

## 3 RESULTS

### 3.1 Baseline characteristics

A total of 381 eligible patients were enrolled in this study, and their median age was 58 years, ranging from 19 to 89 years. Totally 171 (44.9%) patients were male, 266 (69.8%) patients had acral melanoma, and 115 (30.2%) patients had cutaneous melanoma. Of all the patients, melanoma was at the upper extremities in 54 (14.2%) patients, at the trunk in 56 (14.7%) patients, and at the lower extremities in 271 (71.1%) patients. Among the eligible patients, melanoma was of T1 stage in 55 (14.4%) patients, of T2 stage in 91 (23.9%) patients, of T3 stage in 123 (32.3%) patients, and of T4 stage in 112 (29.4%) patients (Table 1).

**TABLE 1:** Comparison of clinicopathological variables between groups with different numbers of sentinel lymph nodes.

	SLN biopsied 1–2 *n* = 216 case (%)	SLN biopsied > 2	Total	*p*‐Value
		*n* = 165 case (%)	case (%)	
Gender				
Female	119 (55.1%)	91 (55.2%)	210 (55.1%)	.991
Male	97 (44.9%)	74 (44.8%)	171 (44.9%)	
Age (median)				
<60 years	114 (52.8%)	88 (53.3%)	202 (53.0%)	.914
≥60 years	102 (47.2%)	77 (46.7%)	179 (47.0%)	
Subtype				
Acral	164 (75.9%)	102 (61.8%)	266 (69.8%)	.003
Cutaneous	52 (24.1%)	63 (38.2%)	115 (30.2%)	
Primary location				
Upper extremity	20 (9.3%)	34 (20.6%)	54 (14.2%)	<.001
Trunk	20 (9.3%)	36 (21.8%)	56 (14.7%)	
Lower extremity	176 (81.5%)	95 (57.6%)	271 (71.1)	
SLNB basin				
Groin	184 (85.2%)	97 (58.8%)	275 (72.2%)	<.001
Axilla	26 (12.0%)	55 (33.3%)	81 (21.3%)	
Popliteal	3 (1.4%)	0 (0.0%)	3 (0.8%)	
Bilateral	1 (0.5%)	6 (3.6%)	7 (1.8%)	
Multi‐basin	2 (0.9%)	7 (4.2%)	9 (2.4%)	
Breslow thickness (T stage)				
0–1 mm	34 (15.7%)	21 (12.7%)	55 (14.4%)	.272
>1–2 mm	55 (25.5%)	36 (21.8%)	91 (23.9%)	
>2–4 mm	72 (33.3%)	51 (30.9%)	123 (32.3%)	
>4 mm	55 (25.5%)	57 (34.5%)	112 (29.4%)	
Clark level				
I	4 (1.9%)	22 (13.3%)	26 (6.8%)	.462
II	13 (6.0%)	1 (0.6%)	14 (3.7%)	
III	13 (6.0%)	12 (7.3%)	25 (6.6%)	
IV	131 (60.6%)	6 (3.6%)	137 (36.0%)	
V	27 (12.5%)	94(57.0%)	121 (31.8%)	
Unknown	27 (12.5%)	30 (18.2%)	57 (15.0%)	
Ulceration				
No	99 (45.8%)	85 (51.5%)	184 (483%)	.271
Yes	117 (54.2%)	80 (48.5$)	197 (51.7)	
SLN status				
Negative	141 (65.3%)	108 (65.5%)	249 (65.4%)	.971
Positive	75 (34.7%)	57 (34.5%)	132 (34.6%)	
Non‐SLN status				
Negative	41 (19.0%)	38 (23.0%)	79 (59.4%)	.161
Positive	18 (8.3%)	5 (3.0%)	23 (17.3%)	
No dissection	17 (7.9%)	14 (8.5%)	31 (23.3%)	
N stage				
N0	141 (65.3%)	107 (64.8%)	248 (65.1%)	.630
N1	45 (20.8%)	31 (18.8%)	76 (19.9%)	
N2	25 (11.6%)	25 (15.2%)	50 (13.1%)	
N3	5 (2.3%)	2 (1.2%)	7 (1.8%)	
TNM stage				
Stage I	57 (26.4%)	38 (23.0%)	95 (24.9%)	.731
Stage II	84 (38.9%)	69 (41.8%)	153 (40.2%)	
Stage III	75 (34.7%)	58 (35.2%)	133 (34.9%)	
Gene mutation				
BRAF	43 (19.9%)	42 (25.5%)	85 (22.3%)	.669
NRAS	27 (12.5%)	15 (9.1%)	43 (11.3%)	
CKIT	14 (6.5%)	10 (6.1%)	24 (63.0%)	
BNK wide‐type	90 (41.7%)	66 (40.0%)	156 (40.9%)	
Unknown	42 (19.4%)	32 (19.4%)	74 (19.4%)	
Adjuvant therapy				
Observation	86 (39.8%)	52 (31.5%)	138 (36.2%)	.135
IFN	50 (23.1%)	39 (23.6%)	89 (23.4%)	
Immunotherapy	65 (30.1%)	59 (35.8%)	124 (32.5%)	
Targeted therapy	9 (4.2%)	13 (7.9%)	22 (5.8%)	
Chemotherapy	1 (0.5%)	0 (0.0%)	1 (0.3%)	
IO + Chemo	4 (1.9%)	1 (0.6%)	5 (1.3%)	
IO + TT	0 (0.0%)	1 (0.6%)	1 (0.3%)	
Radiation	1 (0.5%)	0 (0.0%)	1 (0.3%)	
Relapse pattern (initial)				.435
Regional	34 (15.7%)	19 (11.5%)	53(13.9%)	.778
LN	19 (8.8%)	10 (6.1%)	29(7.6%)	.372
Basin	7 (3.2%)	7 (4.2%)	14(3.7%)	
Other	12 (5.6%)	3 (1.9%)	15(3.9%)	
LR + ITT	11 (5.1%)	7 (4.2%)	18(4.7%)	
Mixed	4 (1.9%)	2 (1.2%)	6(1.6%)	
Systemic	22 (10.2%)	15 (9.1%)	37(9.7%)	.395
Oligo	12 (5.6%)	4 (2.4%)	16(4.2%)	
Multi	10 (4.6%)	19 (11.5%)	29(7.6%)	
In‐transit^a^	18 (8.3%)	11 (6.7%)	29(7.6%)	.543
No relapse	160 (74.1%)	131 (79.4%)	291(76.4%)	

Abbreviations: Chemo, chemotherapy; IFN, interferon; IO, immunotherapy; ITT, in‐transit; LR, local relapse; SLN, sentinel lymph node; SLNB, sentinel lymph node biopsy; TT, targeted therapy.

^a^ In‐transit occurrence overall.

Among patients who underwent SLNB, SLNB was performed on 281 patients (73.8%) within the groin. Of all these patients, 132 (34.7%) patients were pathologically diagnosed with SLN‐positive. Among them, 101 (76.5%) patients received CLND within 1 month, while the others declined. Of 101 patients who underwent CLND, 23 (22.8%) patients were found to have non‐SLN‐positive. The median follow‐up was 24 (range: 5–62) months. Besides, 291 (76.4%) patients had no relapse during the follow‐up period, while 82 (21.5%) patients were identified with local/satellite/in‐transit metastases or regional lymph node metastases and 37 (9.7%) patients with systemic metastases.

### 3.2 Number of biopsied SLNs and positivity rates

As the median number of biopsied SLNs was equal to 2 (range: 1 to 20), patients were assigned into two groups: 216 (56.7%) patients with 1 to 2 biopsied SLNs in group one, and 165 (43.3%) patients with more than 2 biopsied SLNs in group two. Upon Pearson's chi‐squared test and the Fisher's exact test, no significant difference in the clinicopathological variables, such as gender, age, Breslow index, ulceration rate, N stage, TNM stage, gender, the Clark level, gene mutation, and pattern of relapses between the two groups. Of all these patients, 243 (63.8%) patients received adjuvant therapy and no significant difference was found in adjuvant therapies between these two groups (Table 1). As melanoma of acral type was dominant in our country and in which lower extremity was the most common primary tumor location, groin became the most common SLNB basin of these two groups, accounting for 85.2% and 58.8% respectively. The rate of SLN‐positivity was 34.7% in group one and 34.5% in group two, while the rate of non‐SLN‐positivity in the two groups was 8.3% and 3.0% (*p* = .161), respectively. It was revealed that in patients with Breslow >4 mm and N1a status, the rate of non‐SLN‐positive was 40.7% (11/27) in group one, which was higher than that in group two (17.6%, 3/17), but not statistically significant (*p* = .109). Furthermore, there were more cases with acral melanoma (*n* = 164, 75.9%, *p* = .003), more patients with melanoma at the lower extremities (*n* = 176, 81.5%, *p* < .001), and a higher rate of SLNB in groin (*n* = 184, 85.2%, *p* < .001) in group one (Table 1). Besides, 17 (7.9%) patients in group one and 14 (8.5%) patients in group two declined CLND, and no significant difference was detected.

### 3.3 Survival rates

**FIGURE 1:**
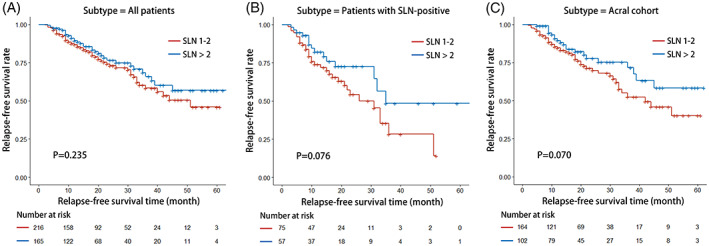
Relapse‐free survival of patients with different numbers of biopsied sentinel lymph nodes (SLNs). (A) All patients; (B) Patients with SLN‐positive; (C) Patients with acral subtype.

**FIGURE 2:**
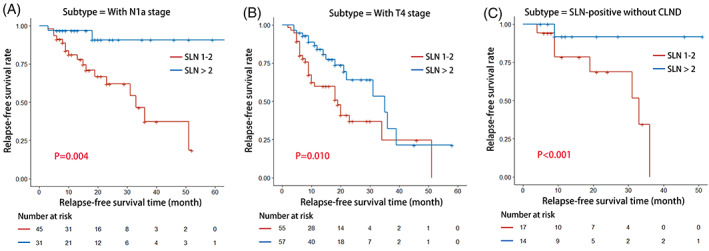
Relapse‐free survival of patients with different numbers of biopsied SLNs in specific subtypes. (A) Patients with N1a stage; (B) Patients with T4 stage; (C) Patients with SLN‐positive but without completion lymph node dissection (CLND).

The Kaplan–Meier method was used to identify the survival data of patients with different number of SLNs. The median RFS of all patients was 46.4 (95% CI: 42.9–50.0) months. The 1‐ and 2‐year RFS rates of SLN‐positive and SLN negative patients were 77.4% and 61.3%, and 93.5% and 81.4%, respectively (*p* = .235). With the log‐rank test between subgroups, the median RFS in group one was 41.9 (95% CI: 37.9–45.8) months, with no significant difference compared with group two (mRFS: 48.6 months, 95% CI: 43.4–53.7 months) (*p* = .235, Figure 1A). Similar results were found in patients with SLN‐positive (*p* = .076) and acral subtype (*p* = .070) (Figure 1B,C). However, a longer RFS was found in group two with Breslow thickness >4 mm (mRFS: 19 vs. 35 months, respectively, *p* = .010), N1a stage (mRFS: 33 months vs. not reached, respectively, *p* = .004), and patients without CLND after SLN‐positive (mRFS: 33 months vs. not reached respectively, *p* = .044) (Figure 2A–C).

## 4 DISCUSSION

In this retrospective study involving Chinese melanoma patients with no nodal disease who received SLNB, identification and retrieval of 3 and more SLNs did not correspond in a different RFS compared to patients with only 1 or 2 SLNs. In patients with 3 or more SLN, there was a trend towards a longer RFS for patients with severe Breslow thickness (>4 mm), N1a stage, and those with SLN‐positive without plan of CLND. This result implied the underling importance of the number of SLNs in melanoma, which was rarely mentioned before.

In melanoma, SLN is mainly identified by using lymphoscintigraphy after injecting radioactive technetium adjacent into the primary lesion, or injecting methylene blue dye preoperatively into four quadrants at derma layer around the primary site. Detection of the SLNs was then finished by using a gamma probe and visual observation of methylene blue dye lymphatic drained into the regional lymph node field. Despite splendid progress in the techniques and expertise of SLN mapping, SLNB still possesses a false‐negative rate of about 10%–20%.^16^, ^17^, ^18^ Indocyanine green is a new method mapping the lymph node without using radioactive tracer and without the risk of tattooing that is associated with the use of blue dye.^19^ However, success rates for SLN identification are insufficient, because they merely indicate the proportion of patients in which at least one SLN was identified and removed, while they do not define the proportion of patients in which all SLNs were identified by accurate preoperative lymphoscintigraphy.^20^

According to the final reports of German Dermatologic Cooperative Oncology Group trial (DeCOG‐SLT) and Multi‐Center Selective Lymphadenectomy Trial II (MSLT‐II), immediate CLND did not bring survival benefits to SLN‐positive patients.^21^, ^22^ With the dominant subtype was acral melanoma in Asian population, Chinese melanoma patients tended to have a higher Breslow index (mean Breslow thickness: 2.7 mm), ulceration rate (51.6%), and especially, a higher proportion of positive SLNs (34.7%) and non‐SLNs (22.8%) than Western patients.^23^, ^24^, ^25^ Thus, whether CLND is still unbeneficial for OS in Asian patients requires validation in a larger sample‐sized study, although it is the question if such a trial will ever be conducted. As Chinese patients always have more tumor burden, higher rates of non‐SLN metastasis and adjuvant systemic therapy in acral melanoma might be less effective than in other cutaneous melanomas, CLND for patients with SLN‐positive is still strongly recommended in daily practice, followed by adjuvant systemic therapy. Patients who rejected CLND were featured by a slight tumor burden in lymph nodes, primary site at shallow layer of skin, and older age. In this study, the rate of CLND in patients with SLN‐positive was 73.8%.

Several studies have concentrated on the relationship between SLN status and prognosis, in which the pathological evaluation and tumor burden in SLN were particularly addressed. However, technical requirements of SLNB still require further attention. To date, some studies showed the number of SLNs‐positive was associated with the survival of patients. Gershenwald et al. demonstrated that 5‐year survival rates ranged from 70% for patients with one SLN and micro‐metastatic lesions to 39% for patients with four or more involved nodes or with nodes that were extensively involved.^3^ However, further study is required to indicate whether the number of biopsied SLNs influences the prognosis of these patients. Generally, the number of SLNs is more than one. A total of 63 377 cases of cutaneous and acral melanoma who received SLNB were recorded in the database of Surveillance, Epidemiology, and End Results (SEER) from 2000 to 2020, of which the median number of SLNs is 2. Similarly, the median number of SLNs was also 2 in the present study, which was in accordance to the results of previous studies.^22^, ^26^ The present study showed that different numbers of biopsied SLNs had no influence on the RFS in the general population, while no significant difference was found in the exact number of positive lymph nodes or the rate of SLNB‐positive between the two groups.

However, patients with >2 SLNs had a longer RFS than those with 1–2 SLNs in T4, N1a group and those who rejected CLND. The underlying reasons might be summarized as follows: (i) identification of more SLNs might be associated with a higher accuracy of estimating N stage, and a lower false‐negative rate; (ii) the median number of SLNs in acral subtype was less than that of the cutaneous subtype, indicating that for acral melanoma, especially in patients with lesions at lower extremities and biopsied in groin, there might be a higher risk of incomplete nodal detection; (iii) for primary lesion site with Breslow thickness >4 mm and a higher rate of SLN‐ positive, evaluation of adequate SLNs might be more critical; and (iv) a higher number of biopsied SLNs‐positive reduced the probability of residual tumor, and lowered the relapse risk in patients who rejected CLND. In addition, the rate of non‐SLN‐positive was 40.7% in cohort with 1–2 SLNs in patients with Breslow >4 mm and N1a stage, which was higher than that of cohort with >2 SLNs (17.6%). This result was in accordance with findings from Rossi et al. that fewer nodes excised was a risk factor for non‐SLNs involvement of metastasis.^12^ This might further explain why SLN‐positive (>2 SLNs) patients without plan of CLND had a significantly longer RFS. As higher SLNs were extracted from the basin, the residual tumor burden in lymph nodes decreased correspondingly. Thus, 3 and more SLNs seem essential for patients with Breslow thickness >4 mm and those with no plan of CLND.

Although the present study provided new insights into the effects of the number of SLNs on the RFS of melanoma patients, its limitations are also noteworthy. Due to the retrospective design of the study, the risk of selection bias is inevitable, indicating the necessity of further prospective study. Furthermore, the effects of different adjuvant therapies on the disease relapse and OS were not assessed. It is noteworthy that targeted therapy might be more efficacious than other adjuvant therapies, such as immunotherapy, during the early period of disease. In addition, data were collected from one single‐center, demonstrating that further multicenter studies are required to indicate the potential influence of the number of SLNs on the prognosis of melanoma patients and to explore its intrinsic mechanism.

## 5 CONCLUSIONS

In conclusion, we found that the number of identified and retrieved SLNs did not significant impact on RFS. In patients with T4 melanomas, N1a melanomas and those that did not undergo a CLND, the prognosis of those with three or more SLNs retrieved seemed to be improved. Three and more SLNs are essential for patients with Breslow thickness >4 mm and those with no plan of CLND.

## AUTHOR CONTRIBUTIONS

**Tu Hu:** Data curation (equal); investigation (equal); writing – original draft (equal). **Yu Xu:** Conceptualization (equal); formal analysis (equal); writing – review and editing (equal). **Wangjun Yan:** Investigation (equal); project administration (equal). **Chunmeng wang:** Data curation (equal); software (equal). **Wei Sun:** Data curation (equal); formal analysis (equal); validation (equal). **YunYi Kong:** Methodology (equal); validation (equal). **Yong Chen:** Conceptualization (equal); project administration (equal); writing – review and editing (equal).

## FUNDING INFORMATION

This research did not receive any specific grant from funding agencies in the public, commercial, or not‐for‐profit sectors.

## CONFLICT OF INTEREST STATEMENT

The authors declare that they have no conflict of interest.

## Data Availability

Not applicable.
